# Cross-sectional entomological monitoring combined with professional qualifications in transition areas for yellow fever and autochthonous malaria in the Atlantic Forest in Rio de Janeiro, Brazil

**DOI:** 10.1590/0074-02760240139

**Published:** 2025-03-17

**Authors:** Claulimara Lopes Moreira, Izabel Cristina dos Reis, Daniel Cardoso Portela Câmara, Tania Ayllón, Mariana Dionizio Machado, Agostinho Cardoso Nascimento-Pereira, Jefferson Pereira Caldas dos Santos, Nathan Burkett-Cadena, Nildimar Alves Honório

**Affiliations:** 1 Secretaria Municipal de Saúde de Macaé, Coordenadoria Especial de Vigilância Ambiental em Saúde, Macaé, RJ, Brasil; 2 Fundação Oswaldo Cruz-Fiocruz, Instituto Oswaldo Cruz, Laboratório das Interações Vírus-Hospedeiros, Rio de Janeiro, RJ, Brasil; 3 Fundação Oswaldo Cruz-Fiocruz, Núcleo Operacional Sentinela de Mosquitos Vetores, Rio de Janeiro, RJ, Brasil; 4 Fundação Oswaldo Cruz-Fiocruz, Instituto de Comunicação e Informação Científica e Tecnológica, Rio de Janeiro, RJ, Brasil; 5 Fundação Oswaldo Cruz-Fiocruz, Programa de Computação Científica, Rio de Janeiro, RJ, Brasil; 6 Universidad Complutense de Madrid, Facultad de Veterinaria, Departamento de Sanidad Animal, Madrid, Spain; 7 Universidade Federal do Maranhão, Departamento de Biologia, Laboratório de Entomologia e Vetores, São Luís, MA, Brasil; 8 Fundação Oswaldo Cruz-Fiocruz, Instituto de Tecnologia em Fármacos, Rio de Janeiro, RJ, Brasil; 9 University of Florida, Florida Medical Entomology Laboratory, Vero Beach, FL, USA

**Keywords:** entomological surveillance, vector-borne-diseases, Culicidae, Atlantic Forest, professional qualifications

## Abstract

**BACKGROUND:**

The Atlantic Forest harbours a rich mosquito assemblage, including vectors for diverse arbovirus. Mosquito species adapt to urban-forest landscape changes, acting as bridge vectors for pathogens.

**OBJECTIVES:**

This study evaluated different collection methods for immature and adult mosquitoes combined with improving field personnel qualifications in a transition area between urbanised and sylvatic environments.

**METHODS:**

Immature and adult mosquitoes were collected from 33 collection points established in urban and peri-urban, sylvatic and transitional areas using different capture methods. During the course, 107 professionals were qualified.

**FINDINGS:**

Vectors (Anophelinae and Culicinae) were dominant in the urban/peri-urban environment (51.49%), followed by the transitional (26.69%) and sylvatic (21.82%) environments. *Aedes* (*Stegomyia*) *albopictus* (Skuse), *Ae.* (*Ochlerotatus*) *scapularis* (Rondani), *Ae.* (*Stg.*) *aegypti* (Linnaeus), *Haemagogus* (*Conopostegus*) *leucocelaenus* (Dyar & Shannon), undetermined *Culex*, *Cx.* (*Melanoconion*) *pilosus* (Dyar and Knab), *Cx.* (*Carrollia*) *urichii* (Coquillett), and *Sabethes* (*Sabethes*) *albiprivus* Theobald were most abundant, with *Ae. albopictus* collected from all ecotopes. Ovitrap provided a robust sample of the immature stages (92.8%), whereas other methods contributed 3.59% of total immatures, but greatest species richness (14 species). For adult mosquitoes, Shannon light trap resulted in greatest abundance (86.16%).

**MAIN CONCLUSIONS:**

The use of varied sampling techniques led to collection of a high mosquito species richness, which, combined with programs for training local professionals, should be an integral part of health surveillance for monitoring the risk of vector-borne diseases.

The Atlantic Forest is the second largest tropical forest in South America, constituting a centre of biodiversity, and is threatened by destructive human activities.^1^ ^,^ ^2^ The original area of the Atlantic Forest comprised around 150 million hectares, covering 15% of the Brazilian territory, although a relict percentage of native forest remains.^3^ The forest extends along the coastline from northeast Brazil to Uruguay and contains approximately 20,000 plant species and more than 1,400 species of terrestrial vertebrates and invertebrates, many of which are endemic. The complexity of the Atlantic Forest derives mainly from the geographic changes observed along its entire extension, which are associated with climatic variations. Consequently, the vegetation distribution in the Atlantic Rainforest is influenced by distance from the ocean, rainfall regimen, and altitude.^4^ ^,^ ^5^ Considered as one of the top 25 global biodiversity hotspots,^6^ the Atlantic Forest is one of the most diverse and threatened areas in the world,^1^ ^,^ ^6^ including a rich mosquito (Diptera: Culicidae) assemblage.^7^ ^,^ ^8^ ^,^ ^9^ Important factors contributing to this diversity are the extension of the Atlantic Forest in latitude, which covers 38º, and altitudinal variations, as the forests extend from sea level to an altitude of 1,800 metres.^10^ In addition, interior forests differ considerably from those of the coast, providing a greater variety of habitats and niches, which allows a multiplicity of habitat options for culicids.^11^

Mosquitoes are well recognized as important historic, contemporary and emerging vectors of human pathogens, due to their roles as biological vectors for different pathogens in humans and domestic animals. This includes the transmission of arboviruses, such as those that cause dengue, yellow fever, chikungunya, and Zika, and of protozoa, such as those that cause malaria, in endemic areas. In South America, sylvatic outbreaks of yellow fever, endemic to the Amazon region, have been described in Brazil, with more than 2,000 confirmed cases and 677 deaths reported between 2016 and 2018,^12^ and which continue to afflict the population, with 327 suspected human cases reported during the 2019/2020 period of monitoring by the Brazilian Ministry of Health.^13^ Rio de Janeiro is considered a highly receptive and vulnerable region for sylvatic yellow fever virus (YFV) transmission.^14^ YFV circulates in forests through its sylvatic culicid vectors and vertebrate hosts. Mosquitos of the genera *Haemagogus* and *Sabethes* are considered the main sylvatic vectors of YFV in the New World,^15^ where *Hg. (Haemagogus) janthinomys* Dyar and *Hg.* (*Conopostegus*) *leucocelaenus* (Dyar & Shannon) act as a primary vector in a large area of the neotropical region.^14^ ^,^ ^16^ In urban environments, the transmission of YFV between humans is maintained through *Aedes* (*Stegomyia*) *aegypti* (Linnaeus) bites from infected mosquitoes.^17^ On the other hand, although previously eliminated over 50 years ago, autochthonous malaria cases have been reported in the Brazilian southeastern states in the Atlantic Forest biome, including Rio de Janeiro State, where efficient malaria vectors were recorded, such as *Anopheles* (*Nyssorhynchus*) *darlingi* Root, *An.* (*Nys.*) *aquasalis* Curry, *An.* (*Nys.*) *albitarsis* s.l., *An.* (*Kerteszia*) *cruzii* Dyar & Knab and *An.* (*Ker.) bellator* Dyar & Knab.^18^ ^,^ ^19^

Landscape has a profound impact on vector-borne pathogens, influencing the assemblages of vectors, potential vertebrate hosts, their interactions and the frequency with which vectors bite humans. Deforestation has a strong effect on vector mosquito species, favouring vectors of human pathogens. It is likely that alterations at the urban-forest interface have a particularly strong impact,^20^ modifying vector-host interactions, and leading to increased contact with sylvatic hosts.^21^ ^,^ ^22^ Some mosquito species probably adapt to these altered habitats moving between sylvatic and urban environments, potentially transporting pathogens between sylvatic amplifying foci and spillover sites. The forest edge can be considered an area where zoonotic diseases can spread from different reservoirs to humans.^22^ Areas with sylvatic YFV transmission, for example, were located near densely urbanised areas where the infestation levels of *Ae. aegypti* and *Ae.* (*Stg.*) *albopictus* (Skuse) is high, which increase the risk of re-emergence of the urban transmission cycle throughout South America, particularly in Brazil.^22^ ^,^ ^23^ ^,^ ^24^ ^,^ ^25^

A comprehensive understanding of this complex situation relies upon accurate methods of quantifying the vector assemblage in diverse environments. This presents a substantial obstacle, given the lack of optimal methods of collecting the complete suite of active vectors in regions, such as the Atlantic Rainforest, that contain dozens genera and hundreds of mosquito species from taxonomically and ecologically diverse groups. While different traps and collection methods are available for immature and adult mosquitoes^26^ ^,^ ^27^ ^,^ ^28^ ^,^ ^29^ a unified method for sampling vectors of zoonotic and anthroponotic pathogens is not available. Sampling methods were chosen according to the target species, life stage, and status.^30^ ^,^ ^31^ Most mosquito species rest outdoors or in natural shelters (*e.g.*, vegetation, tree holes, crab holes, caves, and artificial shelters). The Pan American Health Organization (PAHO) encourages the effective implementation of integrated vector management strategies, emphasizing the importance of situational and entomological analysis. In order to collect the entomological indicators proposed by PAHO, it is essential to use appropriate and validated collection methods. In the present work, we employed several stablished collection methods and introduced new ones essential for obtaining accurate and reliable data on vector populations and their ecology. These entomological indices can also apply some of these specific collection methods, as well as suggest new ones that are essential to obtain accurate and reliable data on vector populations and their ecology. These entomological indices can also be useful in monitoring and evaluating intervention strategies. Additionally, virus monitoring in mosquitoes has recently been integrated, expanding the scope of entomology and requiring new collection methodologies in routine entomological surveillance. This contributes to establishing viral surveillance of vectors in sentinel areas.^27^ ^,^ ^28^ ^,^ ^30^ ^,^ ^31^ The methods more frequently used for outdoor sampling include collection with hand nets, aspirators, drop-net cages in the vegetation, resting traps, or artificial pits. In addition, mosquito larvae can be collected by netting, dipping, and aspirating water using pipettes.^31^ ^,^ ^32^ However, studies using different natural mosquito collection methods, such as bamboo traps or natural traps, are scarce.^33^ ^,^ ^34^ ^,^ ^35^ ^,^ ^36^

In the context of vector-borne diseases, particularly concerning entomology, professional training is essential to implement disease surveillance and preventive and control measures in at-risk areas.^27^ In fact, gaps have been recognised in the health training process of endemic disease control agents (ACE). These gaps encompass the recognition of vector species, collection techniques for surveillance, monitoring, and vector control. ACEs, professionals operating in the community for disease surveillance, prevention, control, and health promotion, in accordance with the Brazilian Unified Health System (SUS) (Law 11.350/2006),^37^ face challenges that emphasise the need for continuous and ongoing training. Emphasising the importance of education that extends beyond knowledge sharing, aiming to engage professionals in everyday reality, enhancing their competencies in routine work, decision-making, particularly in emergency situations, community awareness, and promoting practices that reduce vector proliferation, among others, enabling them to feel adequately empowered and valued.^38^ ^,^ ^39^ ^,^ ^40^

Thus, integrated and continuous entomological surveillance in these areas, including qualification programs for local professionals, should be an integral part of health surveillance and control to monitor the risk of vector-human contact in areas with circulating YFV and other arboviruses.^26^ ^,^ ^27^ ^,^ ^30^ ^,^ ^41^ ^-^ ^44^ The objectives of the present study were to evaluate different collection methods for immature and adult mosquitoes, combined with improving field personnel qualifications in cross-sectional entomological monitoring, in a transition area between urbanized and sylvatic environments with documented circulation and human cases of YFV and malaria, in the Atlantic Forest in the State of Rio de Janeiro, Brazil.

## MATERIALS AND METHODS

**Fig. 1:**
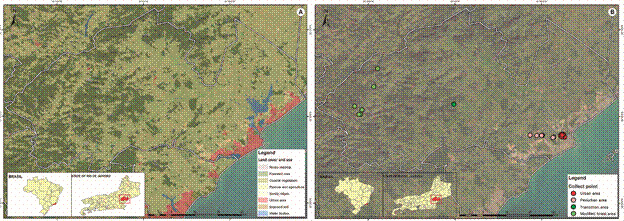
maps of Macaé municipality. (A) Characterization of the collection areas in the municipality of Macaé, Rio de Janeiro. (B) Collection points selected from the urban/peri-urban to rural/sylvatic areas of the municipality of Macaé, Rio de Janeiro.

*Study areas* - The municipality of Macaé, Rio de Janeiro (-22º22’33″ S, -41º46’30 W) belongs to the northern region of Rio de Janeiro State, occupying an area of 1,216.989 km² with an estimated population of 206,728 inhabitants.^45^ Macaé is divided into six districts: Sede, Cachoeiras de Macaé, Córrego do Ouro, Glicério, Frade, and Sana, comprising the mountainous region of the municipality with significant and interconnected remnants of the Atlantic Forest (Fig. 1A). The municipality of Macaé includes 80,510 households and covers 82.3% of the sanitary sewage services. General water distribution networks cover 89.4% of the population, with 8.3% of the households using well or spring water, and 2.3% using other forms of supply. Garbage collection serves 93.6% of households, although regularity of this service is not indicated.^45^

Macaé has a tropical climate, and rainfall is more frequent in winter than in summer. The average annual rainfall was 1,222 mm. The climate classification was Aw (tropical, with dry winter), according to Köppen and Geiger.^46^ Average temperature is 23.3ºC, with February being the warmest month of the year, with an average temperature of 26.0ºC. Relative humidity (RH) is generally high, ranging from lowest in September (76.62%) to highest in December (83.21%) (Climate-Data.org). In terms of land use, analysis indicates significant changes over the past several decades. Data obtained by “Mapping of land use and land cover of the State of Rio de Janeiro” in 2015 at a scale of 1:100,000 (executed in 2017 based on LANDSAT-8 multispectral panchromatic OLI sensor images, carried out by the State Environmental Institute - INEA-RJ) revealed variations in important parameters measured at three different times (1985, 2005, and 2020). In particular, there was a decrease in the forested area from 44,438 ha to 43,461 ha, an increase in the deforested area (6,031 ha in 2020 compared with 2,133 ha in 1985), a decrease in agricultural land, and a decrease in water accumulation areas (lakes, marshes, etc.) from 712 ha to 459 ha.

*Study design* - This work entailed an ecological investigation focused on training and qualification of local professionals for entomological monitoring in an area of yellow fever and autochthonous malaria circulation. A total of 107 health professionals qualified for entomological surveillance by means of trap operation and collection of immature and adult forms in the field as part of a three days (20 h) training program and participation in mosquitoes’ collection in partnership with the Institute of Biodiversity and Sustainability NUPEM/UFRJ - Macaé, Rio de Janeiro.

This entomological survey was carried out along a transect that spanned an anthropogenic gradient, from urban to the sylvatic environments, where circulation of both yellow fever and malaria occurs in the region. On this transect, 33 collection points (Fig. 1B) were randomly selected as follows: (a) 20 properties were selected for trap installation in the peri-domestic in the urban/peri-urban area, including 10 located in Aroeira neighbourhood and 10 in Horto neighbourhood, representing urban and peri-urban areas, respectively. The Aroeira neighbourhood has approximately 6,494 residences, 409 commerce areas, 355 vacant lots, and 186 properties, and is considered one of the most populated neighbourhoods in the municipality of Macaé, while Horto constitutes a neighbourhood with vast vegetated ponds, farms, plantations, and animal breeding (pasture). (b) The Atalaia Municipal Park, with forested areas, was considered the transition area between urban and sylvatic environments and included seven randomly selected collection points, five inside the forest, and two at the park’s edge.

Atalaia consists of rich Atlantic Forest biome, with trails, waterfalls, and wild animals. It has had outbreaks of animal diseases and has a large flow of people from different municipalities; (c) the sylvatic area (modified forested area) covered the District of Sana, an Environmental Protection Area established in 2001 by Municipal Law No. 2172, which encompasses the entire district, and includes six randomly established collection points: two in Cabeceira do Sana, a highly vegetated area with vegetation, wetlands, and animal husbandry, having confirmed cases of malaria and sylvatic yellow fever; three in Arraial do Sana, a more anthropised area with crops, animals, and a wetland cutting through the land; and one in Peito do Pombo, a preserved and wild area with dense preserved vegetation, streams, and isolated from the population of Sana (Fig. 1B).

*Ethics committee* - The present project was approved by the Research Ethics Committee of the Oswaldo Cruz Institute under CAAE:96344718.4.0000.5248.

**Fig. 2:**
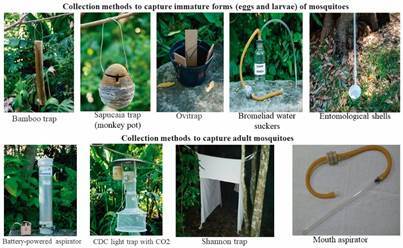
collection methods used for capture of adult and immature forms and eggs of mosquitoes. Own source: credits, Ricardo Schmidt (Instituto Oswaldo Cruz, Brazil).

*Entomological survey methodology* - A pilot study was carried out in August 2018 to characterise the study area, select sampling sites and determine the appropriate collection methods. Collections took place during March, May and September 2019. Diverse methods were used to sample maximum mosquito stadial and species richness. Eggs were sampled using ovitraps, whereas larvae were sampled using bromeliad water suckers, entomological shells (dippers), bamboo traps, and sapucaia (monkey pot) traps. Adults were collected using battery-powered aspirators, Castro capturer, carbon dioxide baited CDC light traps, and Shannon traps (Fig. 2). Ovitraps consisted of 750 mL a black cylindrical container filled with water (with or without leavers) and Eucatex paddles for female oviposition anchored to the vessel using clips.^47^ ^,^ ^48^ Ovitraps remained 15 days each month of the study, and paddles were replaced every week. The methods used for collecting *Haemagogus* spp. eggs were similar to those used by Alencar and collaborators.^11^ Bamboo and sapucaia traps were installed 2-3 m above ground level, remained in the field for 30 days, with replacement of water and collection of immature forms every 15 days. Collection shells (dippers) were used opportunistically to collect Anopheline larvae from accessible larval habitats (ponds and water puddles).^49^

Portable battery-powered aspirators were performed on the day of ovitrap installation, for 20 min at each selected household. CDC light traps, baited with carbon dioxide (dry ice), were installed once per collection month, on the same day as the ovitraps were installed, remaining on for 24 h. Nocturnal mosquitoes were collected using a Castro capturer, consisting of a transparent tube connected to a flexible hose with a smaller tube coated with a screen or fabric as an internal filter, secured with a plastic clamp. Additionally, a Shannon trap was, operated for 3 h (18.00 to 21.00 h) in selected areas.

Different sampling methods were used in each environment due to optimising the methods to quantify vector assemblage, increase collection capacity and improve professional qualification. (A) In Aroeira/Horto (urban/peri-urban) sampling consisted of 20 (one ovitrap per household), portable battery-powered aspirators (five in Aroeira and five in Horto), and one CDC light trap; (B) In Atalaia Municipal Park (transition/forested area) sampling consisted of pipettes and bromeliad water suckers, three bamboo traps and one sapucaia trap, five modified ovitraps, Castro capturer and one Shannon trap; (C) In Sana Environmental Protection Area (sylvatic environment/modified forested area) sampling consisted of modified ovitraps, bamboo, and sapucaia traps, entomological shell (dipper) one CDC light trap and one Shannon trap in selected areas (Table I).

**TABLE I:** Capture methods and/or traps used at different collection points in the municipality of Macaé, Rio de Janeiro

	Larval stage collection methods						Adult stage collection methods			
Locality	Coll. point	Ovitrap	Bamboo trap	Monkey pot trap	Active search	Entomological shell	Active search (Castro capturer)	Aspiration	CDC	Shannon
Aroreira	1									
	2									
	3									
	4									
	5									
	6									
	7									
	8									
	9									
	10									
Horto	11									
	12									
	13									
	14									
	15									
	16									
	17									
	18									
	19									
	20									
Pq. Atalaia	21A									
	21B									
	21									
	22									
	23									
	24									
	25									
Cabeceira do Sana	26									
	27									
Arraial do Sana	28									
	31									
	32									
Vale do Peito do Pombo	29									

*“Active larval search conducted using pipettes and bromeliad suckers, and adult active search included Castro capturer”; modified ovitraps were used at Pq. Atalaia, Cabeceira do Sana, and Vale do Peito do Pombo.

All specimens were collected labelled, properly preserved, and transported to the laboratory. Paddles with eggs were immersed in dechlorinated water and kept at 28 ± 1ºC in the Núcleo Operacional Sentinela de Mosquitos Vetores - Nosmove/Fiocruz, Brazil, until the emergence of aduld mosquitoes. Mosquito identification were performed in the Laboratório de Mosquitos Transmissores de Hematozoários - Lathema/IOC (Brazil) and the Núcleo Operacional Sentinela de Mosquitos Vetores - Nosmove/Fiocruz using the taxonomic keys of Lane^50^ (1953), Arnell^16^ (1973), Consoli & Lourenço-de-Oliveira^51^ (1994) and Forattini^52^ (2002), articles with species descriptions and redescriptions, and comparison with specimens deposited in the “Coleção de Culicidae da Fiocruz” (Fiocruz-CCULI).

*Professional development* - Professional training of health professionals was divided into two modules: “introductory course” and “field practice”. The introductory course on arboviruses and malaria vectors in the municipality of Macaé took place in March 2019 throughout three days in the auditorium of the Institute of Biodiversity and Sustainability NUPEM/UFRJ-Macaé, and included a workload of 20 h. The target audience comprised endemic disease control agents (EDCAs) from Macaé. The average number of participants during the three days was 107. The objective of the course was to present, with theory and practical lessons, the main mosquito vectors of arboviruses and malaria, the main methods for collecting immature forms and capturing adults, in addition to providing an exchange of experiences among managers, EDCAs, education and research professionals. The course was divided into seven topics, covering several components, as shown in Table II. All lectures were prepared and presented by different researchers and collaborators in this project, addressing: (i) the challenges of the epidemiological and entomological scenario of arboviruses in Brazil; (ii) understanding medical entomology and the ecology of its vectors, including yellow fever and malaria competent vectors; (iii) an introduction to the taxonomy of Culicidae; (iv) presentation of various methods of collecting and capturing mosquito vectors; (v) an integrated approach for entomovirological surveillance in arbovirus endemic areas for arboviruses and viral detection; (vi) geoprocessing and (vii) exchange of experience with the EDCAs. The field practice phase consisted of training in mosquito traps and different collection methods and participation in mosquito collection for this research. All experiences and challenges encountered by participants were shared and discussed during their field routines. Practical classes were divided into four stations, called “sharing stands”, grouped into (i) laboratory, (ii) collection and methods of immature forms, (iii) collection and methods of adult forms, and (iv) aspiration. The four groups were interspersed with the stations every 30 min. Each station consisted of an explanation of the different methods and tools, their proper use by the EDCAs, and dialogue from their field experience. The identification of different Culicidae life stages and taxonomic characteristics took place in the didactic laboratory of the institution.

**TABLE II:** Program outline of the Introductory Course on Vectors of Arboviruses and Malaria provided to endemic disease combat agents in the municipality of Macaé, Rio de Janeiro, March 26-28, 2019

Subject	Content topics	Workload (hours)
Challenges of the epidemiological and entomological scenario of arboviruses in Brazil	Arboviruses and emerging and re-emerging diseases; Contributions of medical entomology to the field; Interactions and the factors that influence vector spatial and temporal dynamics.	1
Understanding medical entomology and the ecology of its vectors	Introduction to medical entomology; Order Diptera and family Culicidae; Subfamily Culicinae: genera *Aedes*, *Haemagogus* and *Sabethes*; Subfamily Anophelinae: genus *Anopheles* and subgenera *Nyssorhynchus* and *Kerteszia*.	3
Introduction to the taxonomy of Culicidae	Main morphological characters for the identification of adult mosquitoes; Practice in morphological observation of larvae and adult mosquitoes.	2,5
Presentation of various methods of collecting and capturing mosquito vectors - “Sharing Booth”	Presentation of the main collection methods for immature and adult forms of sylvatic mosquitoes, *Aedes* and *Anopheles*; Practice in the use of the main methods of collection and capture of sylvatic mosquitoes, *Aedes* and *Anopheles.*	2
Integrated approach for entomo-virological surveillance in arbovirus endemic areas for arboviruses: viral detection	Introduction to entomovirological surveillance; Viral detection in adult mosquitoes; Molecular analysis.	1
Geoprocessing and its importance	Introduction to geoprocessing; Mapping and monitoring; Practice: installation of ovitraps and use of GPS.	3,5
Dialogue and exchange of experience with the endemic disease control agents	Presentation of the participants - What do you expect from the course? Presentation and discussion about the master’s degree project; Discussion about work routine, experience and challenges; Evaluation of the course.	7

*Data analysis* - Entomological data were aggregated for the entire study period because of the unbalanced structure of the collection efforts across environments and years, and because we were interested in the ecological aspect of the data (rather than the temporal one). We generated species accumulation curves using individual-based interpolation and extrapolation (up to twice the number of observed individuals in each analysed environment) using the iNEXT R package. Diversity indices, including species richness (S), Simpson diversity (D), Shannom-Wiener diversity (H’) and Sorensen Similarity Index, were calculated to measure mosquito diversity in the different studied environments and in each of the collection methods. Because our preliminary analysis did not meet the assumptions of normality of residuals, we chose to use Kruskal-Wallis tests to compare mosquito diversity (both S and H’) in the different environments (urban/peri-urban, transition, and sylvatic) and collection methods (battery-powered aspirator, bamboo trap, Sapucaia-monkey pot, CDC light trap with carbon dioxide, ovitraps, Castro capturer, and Shannon trap). Additionally, to estimate the number of undetected species in our study, we used the Chao1-bc estimator (which uses the number of observed singletons and doubletons to estimate the number of undetected species).^53^ All analyses were performed using R software (R Core Team), R Studio (RStudio Team) using the SpadeR library^54^ and QGIS 3.22.

## RESULTS

*General* - A diverse mosquito assemblage was collected using the methods outline above, consisting of 50 taxa (26 species and 24 morphotypes) from 14 genera (Table III) comprising 2,572 specimens belonging to two subfamilies (Anophelinae and Culicinae), with Culicinae distributed among six tribes (Aedini, Culicini, Mansoniini, Sabethini, Toxorhynchitini, and Uranotaeniini). The most diverse taxa sampled included *Culex* (12 taxa) and *Wyeomyia* (nine taxa), followed by five taxa each of *Aedes*, *Anopheles*, and *Sabethes*. The remaining genera (*Coquillettidia*, *Haemagogus*, *Limatus*, *Mansonia*, *Onirion*, *Psorophora*, *Runchomyia*, *Toxorhynchites*, *Uranotaenia*) were represented by one or two taxa (Table III). Most specimens (1,977; 77.59%) were subimagoes (1,834 eggs and 143 larvae) and 571 (22.41%) were adults. Of these, it was not possible to identify the species or define the morphotypes for 156 specimens (6.07%).

**TABLE III:** Mosquito specimens collected by taxonomic category, and sampling methods (numbers and percentages) from urban/peri-urban to sylvatic areas conducted in the municipality of Macaé, Rio de Janeiro during the study period

	Larval stage					Adult stage					
Taxonomic category	Ovitrap	Bamboo trap	Sapucaia trap	Active search	Entomological shell	Active search (Castro capturer)	Aspiration	CDC	Shannon	Total	%
*Aedes (Och.) scapularis*						1	2		386	389	15,27
*Aedes (Och.) taeniorhynchus*							5			5	0,20
*Aedes (Och.) terrens*	41					1			3	45	1,77
*Aedes (Stg.) aegypti*	350						4			354	13,89
*Aedes (Stg.) albopictus*	1146			2		6				1154	45,29
*Anopheles (Ano.) fluminensis*									1	1	0,04
*Anopheles (Nys.) albitarsis s.l.*					2					2	0,08
*Anopheles (Ker.) cruzii*					1					1	0,04
*Anopheles (Nys.) evansae*					1					1	0,04
*Anopheles sp.*					4					4	0,16
*Coquillettidia (Rhy.) chrysonotum/albifera*									1	1	0,04
*Culex (Car.) urichii*	62									62	2,43
*Culex (Cux.)*						1	4		7	12	0,47
*Culex (Cux.) nigripalpus*							4			4	0,16
*Culex (Cux.) quinquefasciatus*							2			2	0,08
*Culex (Mel.) pilosus*									87	87	3,41
*Culex (Mel.)*					1					1	0,04
*Culex (Mcx.)*						2				2	0,08
*Culex (Phe.)*					4					4	0,16
*Culex spp.*	68	4		3	18	3	2	2		100	3,92
*Culex sp1.*	1	3								4	0,16
*Culex sp2.*	1									1	0,04
*Culex sp3.*	3									3	0,12
*Haemagogus (Hag.) janthinomys/capricornii*						3				3	0,12
*Haemagogus (Con.) leucocelaenus*	148					2				150	5,89
*Limatus durhamii*				14		3				17	0,67
*Limatus flavisetosus*	11					2				13	0,51
*Mansonia sp.*						2				2	0,08
*Onirion personatum*						1				1	0,04
*Psorophora (Jan.) albipes/albigenu*						2	3		7	12	0,47
*Psorophora (Jan.) ferox*							1			1	0,04
*Runchomyia (Run.) humboldti*							6			6	0,24
*Runchomyia sp.*							1			1	0,04
*Sabethes (Pey.)*			2			2				4	0,16
*Sabethes (Sab.) purpureus*			2							2	0,08
*Sabethes (Sbo.) sp.*						1				1	0,04
*Sabethes (Sab.) albiprivus*		32	24							56	2,20
*Sabethes sp.*				1						1	0,04
*Toxorhynchites (Lyn.)*	3		2							5	0,20
*Toxorhynchites sp.*			3							3	0,12
*Uranotaenia*					14					14	0,55
*Wyeomyia (Spi.) bourrouli/forcipenis*							1			1	0,04
*Wyeomyia (Tra.) aporonoma*						2				2	0,08
*Wyeomyia melanocephala*						1				1	0,04
*Wyeomyia (Spi.) mystes*						2				2	0,08
*Wyeomyia (Pho.) edwardsi*				4						4	0,16
*Wyeomyia (Pho.)*				1						1	0,04
*Wyeomyia (Prl.) confusa*							4			4	0,16
*Wyeomyia sp.1*				1						1	0,04
*Wyeomyia sp2.*						1				1	0,04
Total	1834	39	33	26	45	38	39	2	492	2548	100,00

**Fig. 3:**
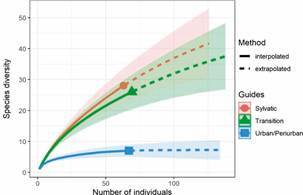
extrapolation curve of species diversity of specimens collected according to the urban/peri-urban, transition/sylvatic, and sylvatic ecotopes in the municipality of Macaé, Rio de Janeiro, from August 2018 to September 2019.

*Assemblage by environment* - The total number of individuals collected from each taxon was segregated into urban/peri-urban, transition, and sylvatic environments. Thus, 1,312 (51.5%) specimens were collected in the urban/peri-urban environment and belonged to seven taxa; 680 (26.7%) specimens were found in the transitional area and were represented by 26 taxa, in the sylvatic environment 556 specimens were collected (21.82%), distributed among 28 taxa. Among the 50 taxa identified, 16 had only one individual collected (singletons, 32.0%), and 20 taxa (not including singletons) had less than ten individuals collected (40.0%). For remaining taxa identified (28.0%), more than 10 individuals were collected. The most abundant species were *Ae.* (*Stg.*) *albopictus* (n = 1,154 individuals, 45.29%), *Ae.* (*Ochlerotatus*) *scapularis* (Rondani) (n = 389, 15.27%), *Ae.* (*Stg.*) *aegypti* (n = 354, 13.89%), *Hg.* (*Con.*) *leucocelaenus* (n = 150, 5.89%), undetermined *Culex* (n = 100, 3.92%), *Culex* (*Melanoconion*) *pilosus* (Dyar & Knab) (n = 87, 3,41%), *Cx.* (*Carrollia*) *urichii* (Coquillett) (n = 62, 2,43%) and *Sabethes* (*Sabethes*) *albiprivus* Theobald (n = 56, 2,2%), these eight groups accounting for 92.3% of the mosquito collected of the total number collected (Tables III-IV). *Ae. albopictus* was the species collected from all ecotopes, while *Ae. aegypti* were collected only from urban environments. The morphotype *Hg. janthinomys/capricornii* were present only in a more preserved sylvatic environment. Species accumulation curves were used to compare species richness between the different ecotopes, as shown in Fig. 3. Thus, the number of species that would need to be captured for a species to be considered representative of that area. Species accumulation curves reached an asymptote in the urban/peri-urban environment, but not in the transition and sylvatic environments, indicating that more species could be found in these locations. The number of taxa identified in the urban/peri-urban environment was matched using the Chao1- bc estimator (mean: [IC95], 7 [7; 8.349]). At least one more species could be collected in the transitional environment (Chao1-bc: 35.320 [27.934; 70.908]) and two more species in the sylvatic environment (Chao1-bc: 35.487 [29.770; 59.659]).

**TABLE IV:** Mosquito specimens collected by taxonomic category and environment in the municipality of Macaé, Rio de Janeiro during the study period

Taxonomic category	Urban / periurban	Transitional / forested	Sylvatic	Total	%
*Aedes (Och.) scapularis*	2	387	-	389	15,27
*Aedes (Och.) taeniorhynchus*	5	-	-	5	0,20
*Aedes (Och.) terrens*	-	18	27	45	1,77
*Aedes (Stg.) aegypti*	354	-	-	354	13,89
*Aedes (Stg.) albopictus*	874	46	234	1154	45,29
*Anopheles (Ano.) fluminensis*	-	-	1	1	0,04
*Anopheles (Nys.) albitarsis s.l.*	-	-	2	2	0,08
*Anopheles (Ker.) cruzii*	-	-	1	1	0,04
*Anopheles (Nys) evansae*	-	-	1	1	0,04
*Anopheles sp.*	-	-	4	4	0,16
*Coquillettidia (Rhy.) chrysonotum/albifera*	-	-	1	1	0,04
*Culex (Car.) urichii*	-	62	-	62	2,43
*Culex (Cux.)*	-	8	4	12	0,47
*Culex (Cux.) nigripalpus*	4	-	-	4	0,16
*Culex (Cux.) quinquefasciatus*	-	-	1	1	0,04
*Culex (Mel.) pilosus*	-	87	-	87	3,41
*Culex (Mel.)*	-	-	2	2	0,08
*Culex (Mcx.)*	-	-	4	4	0,16
*Culex (Phe.)*	2	-	-	2	0,08
*Culex spp.*	71	3	26	100	3,92
*Culex sp1.*	-	1	3	4	0,16
*Culex sp2.*	-	1	-	1	0,04
*Culex sp3.*	-	3	-	3	0,12
*Haemagogus (Hag.) janthinomys/capricornii*	-	-	3	3	0,12
*Haemagogus (Con.) leucocelaenus*	-	9	141	150	5,89
*Limatus durhamii*	-	-	17	17	0,67
*Limatus flavisetosus*	-	13	-	13	0,51
*Mansonia sp.*	-	2	-	2	0,08
*Onirion personatum*	-	-	1	1	0,04
*Psorophora (Jan.) albipes/albigenu*	-	12	-	12	0,47
*Psorophora (Jan.) ferox*	-	1	-	1	0,04
*Runchomyia (Run.) humboldti*	-	6	-	6	0,24
*Runchomyia sp.*	-	1	-	1	0,04
*Sabethes (Pey.)*	-	-	4	4	0,16
*Sabethes (Sab.) purpureus*	-	-	2	2	0,08
*Sabethes (Sbo.) sp.*	-	1	-	1	0,04
*Sabethes (Sab.) albiprivus*	-	1	55	56	2,20
*Sabethes sp.*	-	-	1	1	0,04
*Toxorhynchites (Lyn.)*	-	3	2	5	0,20
*Toxorhynchites sp.*	-	3	-	3	0,12
*Uranotaenia*	-	-	14	14	0,55
*Wyeomyia (Spi.) bourrouli/forcipenis*	-	1	-	1	0,04
*Wyeomyia (Tra.) aporonoma*	-	4	-	4	0,16
*Wyeomyia melanocephala*	-	2	-	2	0,08
*Wyeomyia (Spi.) mystes*	-	1	-	1	0,04
*Wyeomyia (Pho.) edwardsi*	-	4	-	4	0,16
*Wyeomyia (Pho.)*	-	-	1	1	0,04
*Wyeomyia (Prl.) confusa*	-	-	2	2	0,08
*Wyeomyia sp.1.*	-	-	1	1	0,04
*Wyeomyia sp2.*	-	-	1	1	0,04
Total	1312	680	556	2548	100,00
% ecotones	51,49	26,69	21,82	100,00	-

The Alpha diversity analysis showed that urban/periurban exhibited the lowest species richness (n = 7) and diversity (D = 0.480; H’ = 0.841), while transitional/forested and sylvatic habitats had high species richness (n = 26 and n = 28, respectively) and diversity (D = 0.645; H’ = 1.646 and D = 0.742; H’ = 1.844, respectively). Similarity (Sorensen Index) analysis showed that transitional/forested and Sylvatic habitats shared a high degree of species similarity (0.296) with a greater number of shared species (8). In contrast, urban/periurban had low similarity to transitional/forested (0.182) and Sylvatic habitats (0.114), and few shared species (2-3).

**Fig. 4:**
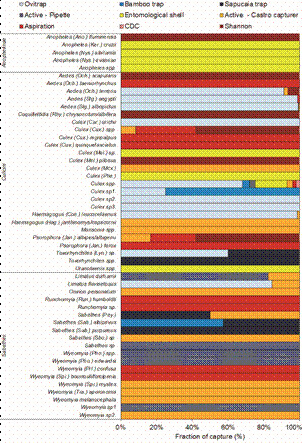
patterns of mosquito group and species captures across collection methods. Methods of sampling immatures are shades of pink and methods of sampling adults are shades of blue. Total number of specimens per species are provided in parentheses.

*Collection methods / traps* - In general, little correspondence could be observed of sampling methods deployed on higher taxonomic groups (subfamily, tribe) with the exception of anophelines, which were overwhelmingly sampled in the larval stage, using entomological shells (dippers). Culicines were mainly sampled in the egg and adult stages, although some taxa (subgenus *Phenacomyia* of *Culex* and *Uranotaenia*) were only sampled as larvae. Sabethines were mainly collected as adults (Castro collectors and aspirators) and larvae, whereas *Limatus flavisetosus* was collected almost exclusively using ovitraps. Among *Wyeomyia*, three taxa were only collected as larvae and six species were only found as adults. Variations in mosquito collection rates using these collection methods are shown in Fig. 4.

Among methods for collecting immature stages, ovitrap represented the collection method with the highest counts of specimens collected (n = 1,834, 92.77%), including 1.494 eggs and 340 larvae, with a richness of 11 taxa identified. Active search in natural habitats, such as bromeliad water suckers and entomological shells, presented the second highest counts (n = 71, 3.59%) and the highest species richness (14 taxa). Bamboo trap and Sapucaia trap sampled 39 individuals (1.97%) of three different taxa, 33 individuals (1.67%) of five taxa, respectively. Regarding the methods aimed at the collection of adult stages, Shannon light trap produced the highest counts (n = 492, 86.16%) of mosquitoes collected from seven different taxa. Battery-powered aspiration and direct aspiration with Castro capturer yielded 39 (6.83%) and 38 (6.65%) individuals, respectively, with Castro capturer yielding highest species richness (19 specimens versus 13 species collected with a battery-powered aspirator). CDC light trap collected just two individuals from one taxa (0.35%). Undetermined *Culex* was collected using all available collection methods, except for the Sapucaia trap, whereas *Ae. albopictus* were recorded using active and other search techniques (Castro capturer, ovitrap and natural breeding sites). In contrast, mosquitoes of the genus *Wyeomyia* were sampled in active searches in natural breeding sites with oral aspiration (Castro capturer), and battery-powered aspirator. Sabethini were collected in bamboo traps, natural larvitraps, and by active search for adults with oral aspiration (Castro capturer). The five identified anopheline species were collected using the shell method only.

Finally, in this study, there was no significant difference in species richness (Kruskal-Wallis χ² =2.94, df = 2, p-value = 0.2299) or in the Shannon diversity index (Kruskal-Wallis χ² = 2.0022, df = 2, p- value = 0.3675) between environments. There were also no significant differences in species richness (Kruskal-Wallis χ² = 8.9306, df = 7, p-value = 0.2577) and the Shannon diversity index (Kruskal - Wallis χ² = 10.375, df = 7, p-value = 0.1683) between the collection methods used.

*Professional development* - The ACEs received comprehensive theoretical and practical training on the main mosquito vectors of arboviruses and malaria and techniques for collecting immature and adult forms of these vectors. The combination of theory and practice was essential to ensure that participants not only understood the collection methods, but also learned how to apply them effectively in the field. Open dialogue and reflection were encouraged, allowing participants to reconsider the proposed approaches and refine their expectations for the training process. This exchange experiences between the ACEs, managers and all course collaborators was a fundamental part of the course, allowing for a collective discussion that culminated in the construction of an Entomological Operational Procedure (POE) and its practical application.

## DISCUSSION

In this study, collection methods and/or traps were used in three different ecotopes within the Atlantic Forest in Rio de Janeiro State. In addition, it integrates a professional qualification program through the training of EDCAs to implement suitable collection methods for the monitoring and surveillance of the circulation of mosquito species associated with arboviruses, such as YFV, and vectors of other important pathogens, *e.g.*, malaria.

Based on the collection methods used, 2,548 specimens were collected, with the majority being immature species and a quarter represented by adult forms. Specimens from two subfamilies, Anophelinae and Culicinae, were identified, the latter included the tribes Aedini and Sabethini, which constitute important vectors in both sylvatic and urban cycles of yellow fever. The different methods used for the collection of immature and adult forms were tested for specificity in the study area. In our study, regarding the methods used in the entomological survey, carbon dioxide-baited CDC light traps performed poorly and contributed little to the investigation of yellow fever suitable vectors in the region. The natural traps were efficient, with Sapucaia being the most suitable for collecting diverse *Sabethes* mosquitoes.^35^ This is probably due to the habit of this genus to seek natural breeding with the least possible opening to external environment sites for oviposition.^33^

Adapted ovitraps were very efficient in collecting eggs from *Haemagogus* mosquitoes, as reported.^11^ *Hg. leucocelaenus* was the species most frequently collected in the ovitraps, a finding expected because of the bias of the trap and the species tolerating anthropogenic alteration of the breeding sites. This species, which can disperse for up to 6 km, is adapted to both secondary forests and environments with notable anthropogenic alterations.^55^ ^,^ ^56^ For decades, *Hg. leucocelaenus* is considered a secondary vector in the transmission of YFV. However, in the southeast and south regions, *Hg. leucocelaenus* is considered a primary vector, since its abundance is higher than that of *Hg. janthinomys/capricornii* in these regions,^57^ being found naturally infected since the first studies in the 1930s and subsequent years.^14^ ^,^ ^58^ ^,^ ^59^

Collections of adult forms of wild mosquitoes showed that the active search through oral aspiration with the aid of Castro capturer was more specific, capturing more anthropophilic mosquitoes, such as *Hg. janthinomys/capricornii*. Importantly, it was not possible to differentiate between these two species for female characteristics, and because only females were captured, specific determination was impractical. Both sylvatic species have been found naturally infected with YFV in Brazil, including the municipality of Macaé, in Rio de Janeiro.^14^ ^,^ ^60^ ^,^ ^61^ ^,^ ^62^ Castro capturer showed the highest richness among all methods used in this study. The collection of different species of anophelines was success ful using entomological shells (dippers) and Shannon traps. However, the sampling effort in bromeliads was not sufficient to identify anophelines in this natural breeding ground. Nonetheless, epiphytic bromeliads > two meters were not explored. Several studies conducted in Rio de Janeiro have already identified some anopheline species, including *An. aquasalis*, *An. albitarsis*, *An. bellator* and *An. cruzii*, which are distributed in almost all municipalities along the state coastline, except for *An. albitarsis* s.l., which is present throughout almost the entire state.^18^ ^,^ ^19^ On the other hand, *Ae. albopictus* were found in all the ecotopes in this study. This constitutes a concern, as this vector is susceptible to YFV infection, colonises both natural and artificial breeding sites, and can act as a bridge in both YFV transmission cycles, and has recently been found in neglected and densely urbanised areas in Rio de Janeiro.^22^ ^,^ ^42^ ^,^ ^63^ ^,^ ^64^

Regarding the areas selected for the study, three different environments were selected, urban/peri-urban, transition and sylvatic areas. The transition area was located halfway between the urban/peri-urban and sylvatic areas and constituted a preserved area characterized by rich Atlantic Forest vegetation, trails, waterfalls, wetlands, and animal husbandry, providing a favourable area for the development of multiple species of mosquitoes. The most sylvatic area is found in Sana which, despite having highly anthropised environments (*e.g.*, pasture and urbanisation), still has important remnants of the Atlantic Forest, including waterfalls and wells formed by the various streams in the region, as in Peito do Pombo. The species richness analysis in this study showed that in urban/peri-urban areas, the mosquito species richness obtained was close to reality. However, in the transitional environment, it would have been necessary to capture at least one more mosquito species, and in the sylvatic area, two more species. Although there were no significant differences when comparing species richness, either between ecotopes or between collection methods, the recording of species is important for the identification of mosquitoes that are potential vectors of YFV and malaria in the municipality of Macaé, Rio de Janeiro.

The results obtained in this study highlight the importance of qualifying local health professionals for cross-sectional entomological monitoring using different collection methods in the surveillance of arbovirus- and malaria-transmitting mosquitoes. Thus, the qualification of local personnel in entomological surveillance and the use of different mosquito trapping methods, combined with research, contribute to enhanced disease management and arbovirus surveillance programs. Surveys with several sampling methods tend to record higher species richness than those with few or single sampling methods.^65^ ^,^ ^66^ In agreement, the sampling methods used in this study allowed the capture of a wide diversity of mosquitoes, many of which recognised YFV and malaria vectors, which led to the reinforcement of surveillance systems to monitor the circulation of arboviruses, such as YFV or malaria, in this region.^64^ ^,^ ^67^ However, the only method in which satisfactory results were obtained for the collection of anophelines was the entomological shell and the Shannon trap, but positive collection results were not obtained with other effective methods such as protected human attraction and human baited mosquito trap (MosqTent), demonstrated to work effectively for this species, thus emphasising the need to expand the collection area and collection times for this species.^68^

Entomological survey studies provide information on habitat preferences for several mosquito species, many of which lack information on biology, ecology, and distribution in the literature.^43^ ^,^ ^63^ Further studies evaluating the distribution of mosquito species in heterogeneous landscapes should be prioritised to understand the possible role of each species in disease transmission. The use of different collection methods is also of great importance in evaluating the positivity and density indicators of each species, which can be crucial during inter-epidemic and epidemic periods, with the potential to further enhance entomological and virological surveillance in public health.^26^ ^,^ ^69^

The importance of characterising these environments for entomological surveillance and implementing efficient collection methods should be emphasised to predict the emergence of epidemics. Moreover, qualification of professionals for entomological monitoring in vulnerable areas is reflected in the priority activities defined by the WHO for 2017-2030, enhancing “National and regional institutional networks to support training and/or education in public health entomology and technical support established and functioning”.^70^ Furthermore, as a result of the professional qualification program, two teaching materials called “Entomological Operational Procedures for yellow fever^71^ and malaria vectors^72^ were collectively produced with EDCAs, in order to consolidate the knowledge and practices accomplished throughout the course. In fact, this comprehensive perspective on entomological surveillance and the use of different collection methods can greatly increase the knowledge of the bionomics, ecology, and distribution of the species and, with necessary support, reveal the unknown taxonomic aspects of these species. Considering this as a training-oriented study, further research involving a longer period and including additional collection points at other study sites is needed.
